# Electronic parameters in cobalt-based perovskite-type oxides as descriptors for chemocatalytic reactions

**DOI:** 10.1038/s41467-020-14305-0

**Published:** 2020-01-31

**Authors:** Johannes Simböck, M. Ghiasi, Simon Schönebaum, Ulrich Simon, Frank M. F. de Groot, Regina Palkovits

**Affiliations:** 1 Chair of Heterogeneous Catalysis and Chemical Technology, RWTH Aachen University, Worringerweg 2, 52074 Aachen, Germany; 2 Center for Automotive Catalytic Systems Aachen, RWTH Aachen University, Aachen, 52074 Germany; 3 Inorganic Chemistry and Catalysis, Debye Institute for Nanomaterials Science, Utrecht University, Universiteitsweg 99, 3584 CG Utrecht, Netherlands; 4 Institute of Inorganic Chemistry, RWTH Aachen University, 52074 Aachen, Germany

**Keywords:** Environmental sciences, Chemistry, Energy science and technology, Materials science

## Abstract

Perovskite-type transition metal (TM) oxides are effective catalysts in oxidation and decomposition reactions. Yet, the effect of compositional variation on catalytic efficacy is not well understood. The present analysis of electronic characteristics of B-site substituted LaCoO_3_ derivatives via in situ X-ray absorption spectroscopy (XAS) establishes correlations of electronic parameters with reaction rates: TM *t*_*2g*_ and *e*_*g*_ orbital occupancy yield volcano-type or non-linear correlations with NO oxidation, CO oxidation and N_2_O decomposition rates. Covalent O 2p-TM 3d interaction, in ultra-high vacuum, is a linear descriptor for reaction rates in NO oxidation and CO oxidation, and for N_2_O decomposition rates in O_2_ presence. Covalency crucially determines the ability of the catalytically active sites to interact with surface species during the kinetically relevant step of the reaction. The nature of the kinetically relevant step and of surface species involved lead to the vast effect of XAS measurement conditions on the validity of correlations.

## Abstract (web summary)

Design of efficient catalysts requires understanding the decisive electronic parameters for catalytic efficacy and their dependence on elemental composition. Here, the authors report covalency as suitable descriptor of perovskite-type transition metal oxides as chemo-catalysts.

## Introduction

Thermal stability and inherent catalytic activity have inspired research on perovskite-type oxides (LaMeO_3,_ Me = transition metal) as low-cost replacement for platinum group metals for several decades now^1^. Co-based perovskites in particular are among the most active, for instance with superior NO oxidation activity compared with a benchmark Pt/Pd-based diesel oxidation catalyst^2^ and low overpotentials in oxygen reduction reaction (ORR)^3^ and oxygen evolution reaction (OER)^4^, key reactions of catalytic water splitting. Further catalyst development, however, requires more detailed knowledge of the decisive electronic parameters for catalytic efficacy and their dependence on elemental composition. Recent reviews show the vast number of studies on the effects of substitution and synthesis methods of perovskite-type oxides on their catalytic efficacy in both electrocatalysis^5^ and heterogeneous chemocatalysis^6^.

Redox properties such as transition metal (TM) oxidation states, oxygen vacancy formation, or binding strength of reactants are commonly cited characteristics in the assessment of catalytic efficacy of perovskite-type oxides substituted with different TM^1,7^. These characteristics are a multifaceted expression of electronic states of the bulk and/or surface sites of the catalyst as Hwang et al.^5^ recently emphasized by their correlation of TM 3*d*-orbital occupancy with catalytic rates in the oxidation of NO, CO, or hydrocarbons. Volcano-type plots equally result in the correlation of overpotential in OER^3^ and ORR^8^ and *d*-orbital occupancy, which highlights parallels in electronic parameters between heterogeneous chemocatalysis and electrocatalysis. Mueller et al. further emphasized the relevance of electronic states related to O^2−^ in addition to those of TM cations near the Fermi-level in the interaction with adsorbate molecules in electrochemical reactions^9^. The sole consideration of TM states is therefore an incomplete representation of the relevant charge distribution in perovskite-type oxides, which disregards the covalent TM–O interaction in particular^5^. Information on covalent binding and band structure such as charge-transfer energy is accessible through combination of X-ray emission spectroscopy, X-ray photoelectron spectroscopy (XPS)^10,11^ or X-ray absorption spectroscopy (XAS) at the O K-edge^12^, and is particularly relevant for catalysis when performed in near-ambient conditions. XAS at the TM L_2,3_-edge complements the approach by providing information on spin^13^ and oxidation state of the TM^9,14,15^. Based on these techniques, Suntivich et al. analyzed the binding situation in perovskite TM oxides in detail in terms of TM 3*d*-O 2*p* hybridization, i.e., TM–O covalency, which was quantified and found to describe the overpotential of perovskite-type oxides in both ORR^3^ and OER^12^. Charge-transfer energy was identified as relevant descriptor for trends in overpotential and other parameters for electrocatalytic efficacy as well^4^.

While these correlations are established in electrocatalysis, covalency and *d*-orbital occupancy have not been examined as descriptor in chemocatalysis in detail, particularly with regard to implications of different reaction characteristics or the effects of variable Co oxidation or spin states^13^. A variety of reaction characteristics in chemocatalysis evolves from reaction conditions and reducing or oxidizing nature of the reactants involved^7^. Knowledge of the reaction-specific reactant–catalyst interaction is thus a vital prerequisite for the adequate choice of experimental conditions during in situ material characterization for descriptor analysis. Existing descriptors for chemocatalytic reaction rates do not reflect the complex-binding situation of mixed elemental and orbital character in perovskite-type oxides: formal *d*-orbital occupancy does not reflect covalent bonding and the contribution of O atoms to catalytic activity^5^, and depends strongly on Co oxidation and spin states. The work function of the catalyst material is only reported as descriptor for N_2_O decomposition^16^ as it does not capture the complexity in the electronic structure of the catalyst. A reliable descriptor for comparative materials characterization across singular studies further requires the analysis of surface-specific reaction rates, which are able to reflect different reaction conditions, partial pressures for example, and catalyst characteristics such as varying surface area or TM abundance. A correlation of covalency and surface-specific reaction rates in substituted TM oxides, however, is absent to the best of our knowledge. Efficient catalyst design relies on thorough analysis of descriptors and specific effects that results from the nature of the individual reaction. The ideal descriptor then may become comparable across different reactions, crystal structures, and catalyst compositions of TM oxides.

Here we use quantitative deconvolution of in situ XAS at the TM L_2,3_-edge and O K-edge of B-site substituted LaCoO_3_ derivatives to identify the effect of substitution on electronic parameters and to correlate the latter with surface-specific reaction rates in CO oxidation, NO oxidation, and N_2_O decomposition. This analysis thus considers the significant impact of the variability of Co oxidation and spin states^13^ in LaCoO_3_ and its derivatives. The trend of electronic parameters with reaction rates is highly dependent on XAS measurement conditions in terms of reducing or oxidizing conditions, which is particularly relevant for the choice of experimental conditions during XAS in any study with regards to chemocatalysis. Covalency stands out among the electronic parameters as a linear descriptor for the reactions. The correlations with reaction rates are interpreted individually based on the respective mechanistic understanding and prevalent XAS measurement conditions to rationalize varying outcomes in correlations depending on the catalyst state during the kinetically relevant step. This relation of electronic parameters in perovskite-type oxides with turnover rates in three separate reactions provides fundamental insight into the complexity of substitution effects on catalytic rates. The identification of covalency as suitable descriptor contributes to a better understanding of required catalyst design for improved catalysts. The implications of experimental conditions during XAS on the correlations in different reactions, which require further detailed analysis, underline the relevance of mechanistic understanding and appropriate choice of XAS experimental conditions for the analysis of catalyst design principles for different reactions. While this study is confined to perovskite-type oxides, a brief comparison of N_2_O decomposition on TM oxides with varying crystal structure reveals parallels in different electronic descriptors reported in literature. This similarity of relevant electronic parameters among various TM oxides illustrates the potential contribution of a generalizable descriptor, possibly covalency, to the understanding of varying catalytic efficacy of substituted TM oxides.

## Results

### Cobalt oxidation and spin states

Phase-pure perovskite-type oxides (Supplementary Fig. 1) of the composition LaCo_0.8_X_0.2_O_3_ (LCX-82) with X = Al, Ni, Zn, or LaCo_1 − *x*_Al_*x*_O_3_ with *x* = [0–0.8] (LaCo_0.8_Al_0.2_O_3_ = LCA-82, for example) were synthesized via a citric acid (CA) route as described in the “Methods” section. Zn and Al substitution leads to a change in unit cell symmetry from the rhombohedrally distorted crystal structure of LaCoO_3_ (LC), space group R-3c, to the ideal cubic perovskite structure, space group Pm-3 m (Supplementary Table 1). Co L_2,3_-edges measured in ultrahigh vacuum (UHV) or in O_2_ presence (0.37 kPa) at 623 K were analyzed to determine spin and oxidation state of the Co cations at different conditions (see “Methods” section and Supplementary Note 2).

All TM cations in the samples are octahedrally coordinated, as is expected for a phase-pure perovskite structure. Al^3+^ and Zn^2+^ are present in their stable valence in the conditions in this work and affect the material characteristics through their interaction with the remaining Co–O substructure as we present in the following. The comparison of L_2,3_-edge spectra in this study to references confirms the presence of Co^2+^ in stable high-spin (HS) state^14^, Ni^3+^ in the low-spin (LS) configuration^17^ and different spin states of Co^3+^. Spin states of Co^3+^ in LaCoO_3_ have been subject to extensive debate in literature^13,18^, as Co^3+^ may occur in the LS ground state, the first excited HS state, or excited states with intermediate spin^19^. Obtained spectra are equivalent to previous results related to a mixture of LS and HS states and spin state abundance was thus determined by a least squares fit of a linear combination of two reference spectra^13^ representing a pure LS configuration and a mixed LS–HS state. HS states become increasingly populated with temperature in both UHV and O_2_ presence as excitation becomes more prevalent (Supplementary Table 2).

**Fig. 1:**
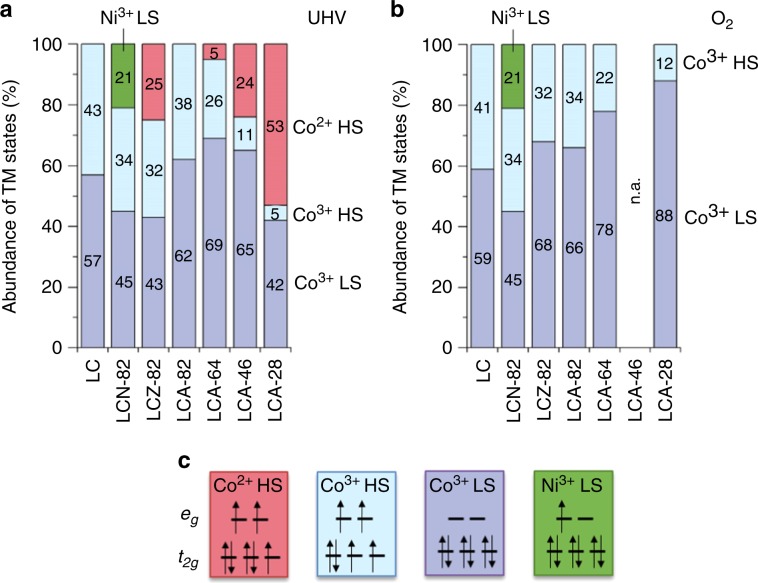
Abundance of transition metal oxidation and spin states. Abundance of transition metal (TM) states as derived from X-ray absorption spectroscopy at the Co L_2,3_-edges at 623 K (**a**) in ultrahigh vacuum (UHV) and (**b**) in 0.37 kPa O_2_. **c** Electronic configuration of TM 3*d* orbitals of abundant, relevant electronic states. Spin states are denoted for high-spin (HS) and low-spin (LS) state.

Relative differences in spin state abundance in the catalysts in this study are similar across all temperatures, the discussion of spin states here is therefore limited to the results at 623 K. Co^3+^ LS population increases significantly in either UHV and O_2_ presence with a higher degree of Al substitution (Fig. 1), which manifests in the more significant high-energy shoulder in the L_3_-edge (781.7 eV, Supplementary Fig. 3a, b) as well, as reported previously^20^. In UHV conditions, Zn^2+^ and Ni^3+^ substitution hardly affects the fraction of Co^3+^ HS to LS states. In O_2_ presence, Zn^2+^ substitution yields an increase of Co^3+^ LS state abundance compared with LC, while no significant change is observed for Ni^3+^ as substituent in terms of the ratio of Co LS and HS state abundance. Spin state abundance affects the electronic interaction between Co 3*d* and O 2*p* states in the CoO_3_ substructure^21^, which consists of vertex-connected CoO_6_ octahedra, analogous to the ReO_3_ structure. Electronic states located at La are lower in energy and their effect on electron density in TM *d* orbitals near the Fermi-level is negligible^22^. The substitution of Co^3+^ by Al^3+^ increases the average electron density in the residual Co–O substructure because of the different inductive effect of the less electronegative substituent Al^21^ and a larger overlap integral of O 2*p* with Co 3*d* orbitals than with much less expansive Al 3*p* orbitals. The increased electron density in the Co–O substructure implicates a stronger electron–electron repulsion in the interaction of O 2*p* and Co 3*d* states and therefore an increased crystal-field splitting energy Δ_CF_ in the octahedral coordination. Given the constant nature of the spin coupling energy *J*_S_, larger Δ_CF_ induces higher relative abundance of LS states compared with LC, an effect that increases with Al substitution degree. In addition, a negative cooperative effect between LS and HS states adds to the stabilization of LS states upon Al substitution^23^. Zn, like Al, is less electronegative than Co and does not act as significant electron density acceptor with its closed *d*^10^ configuration, while Ni^3+^ (*d*^7^) is similar to the Co^3+^ configuration in its effect on electron density distribution. As a result, increased LS abundance occurs for the substitution of Co^3+^ by Zn^2+^ but is absent for Ni^3+^ substitution.

The abundance of Co oxidation states does not vary significantly for measurements at different temperatures (Supplementary Table 3) lower than the pretreatment in UHV at 673 K. Nonsubstituted LC retains the Co^3+^ oxidation state at all conditions as L_2,3_-edge spectra agree with literature data^14^, but Co^2+^ is present in UHV for Zn substitution and high degrees of Al substitution and are most significant in LaCo_0.2_Al_0.8_O_3_ with more than half of all Co cations reduced to Co^2+^. Low-degree substitution of Co by either Al or Ni does not significantly affect reducibility of Co^3+^. Ni remains trivalent corresponding to a Ni L_2_-edge peak position at ~871 eV^24^ (Supplementary Fig. 3c). Introduction of O_2_ (0.37 kPa) into the measurement chamber causes complete reoxidation to Co^3+^ in all catalysts that were partially reduced in UHV. The increased reducibility at high substitution degrees of Co^3+^ with Al^3+^ is attributed to the electronic effect elaborated for variation of spin states: different electronegativity of the cations and lower overlap of Al 3*p* and O 2*p* orbitals causes increased electron density in the remaining Co–O hybridized states. Significant Al substitution degrees and the related enhanced electron density in the CoO_3_ substructure facilitates the reduction of Co^3+^ with concurrent formation of oxygen vacancies as the overlap integral of O 2*p*–Co 3*d* orbitals increases as we derive in the analysis of covalency below. Electron density transfer from O 2*p* into Co 3*d* states is thus more facile for high degrees of Al substitution and results in increased Co^3+^ reducibility. Zn^2+^ as substituent affects the stoichiometry of the oxide, which induces a more facile formation of oxygen vacancies with concurrent reduction of a vicinal Co^3+^ ion.

The abundance of oxygen vacancies was calculated using the oxidation state data of the TMs and considered Zn^2+^ content for LCZ. While most catalysts, corresponding to their low reduction degree, had stoichiometric oxygen content (Supplementary Table 4), LCZ-82 had the most significant nonstoichiometry with δ = 0.20 in LaCo_0.8_Zn_0.2_O_3−δ_. The varying oxygen content was considered in the determination of electronic parameters, where applicable.

### Electronic parameters and their intercorrelation

**Fig. 2:**
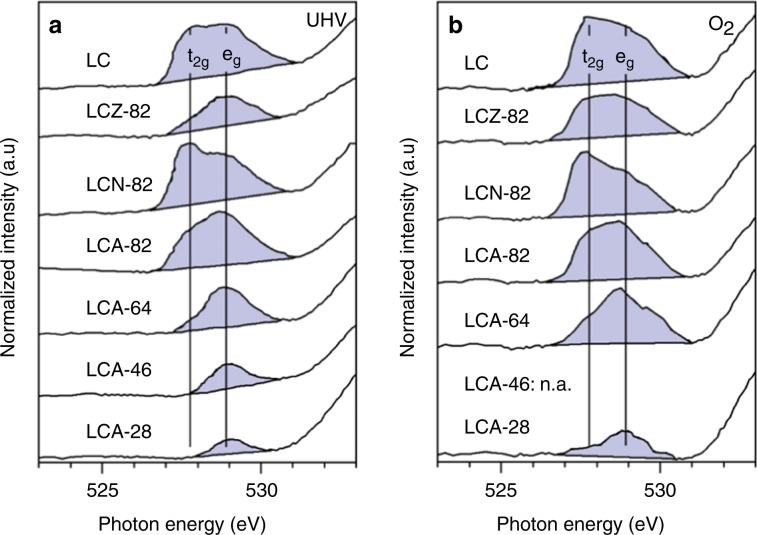
Excerpt of catalyst O K-edge spectra. The shaded area marks feature A of the O K-edge, which is related to O 2*p*–TM 3*d* hybridized states at 623 K (**a**) in ultrahigh vacuum (UHV) and (**b**) in 0.37 kPa O_2_. See Supplementary Fig. 4a, b for full scale O K-edges. Vertical lines mark the estimated peak centers of features in the O K-edge of LC that relate to hybridization of O 2*p* with Co^3+^ *t*_2g_ and *e*_g_ orbitals, respectively.

X-ray absorption at the O K-edge of TM oxides measures excitation of O 1*s* core electrons into O 2*p* states that are unoccupied because of hybridization with cationic states. The related electron density transfer from O 2*p* into cationic states results in different features in the spectra that are addressed in Supplementary Note 3. Feature A, which relates to O 2*p*–TM 3*d* hybridized states (~527–531 eV), can be qualitatively distinguished into hybridization of O 2*p* with *t*_2g_ or *e*_g_ states of Co^3+^ at 527.8 eV or 529.0 eV, respectively^10^ (Fig. 2). The spectral intensity related to hybridized, unoccupied Co^3+^ *t*_2g_ states that occur in HS states decreases relative to the e_g_ feature as Al content increases. The variation in spectral intensity is convoluted by a shift in energy for respective states related to the Co^2+^ HS states in the UHV measurements but is still in agreement with the increasing abundance of LS states, and thus fewer unoccupied *t*_2g_ states, with Al substitution degree as determined in Co L_2,3_-edge analysis. The minor changes in spin states for LCZ compared with LC are also qualitatively reflected in the respective intensity change, while the interaction with unoccupied Ni 3*d* states in LCN appears at approximately the same energy as for Co^3+^ *t*_2g_ states^12^.

Suntivich et al. established the area of feature A in the O K-edge as quantitative measure for the hybridization of O 2*p*–TM 3*d* orbitals, i.e., covalent electron density transfer from O^2−^ to the TM cation^12^. Non-TM cations affect this interaction and the electron density in the Co–O substructure as described in the previous section. The spectral intensity of feature A requires normalization for the abundance of TM cations and oxygen content. Further normalization is required for the abundance of unoccupied 3*d* states (holes) and for the different spectral contribution of *e*_g_ and *t*_2g_ holes that was approximated to a ratio of 4:1 based on the calculated difference in transfer integrals^25^. Experimental results of LaFeO_3_^26^ and TiO_2_^27^ for example, however, disagree with a ratio of 4:1. Instead, when the number of *t*_2g_ and *e*_g_ orbitals (*t*_2g_: 3; *e*_g_: 2) and the respective interactions (*t*_2g_: 2; *e*_g_: 1) is taken into account in addition to the difference in transfer integrals of *e*_g_ and *t*_2g_, a ratio of 8:6 for *e*_g_:*t*_2g_ spectral contribution is the result, which in first approximation fits the results in the abovementioned material examples. The multiplicity of orbitals is relevant because a hole in the solid-state electronic structure is not located in one 3*d* orbital but is distributed among the degenerate orbitals that each interact with the O 2*p* states. The average number of unoccupied states in *e*_g_ and *t*_2g_ states from all TM spin and oxidation states is used for normalization, yielding a covalency factor:

1 $$F_{{\rm{cov}}}\,=\,\frac{{I_{\rm{A}}}}{{\sum\limits_i {x_i\left( {h_{{\rm{e}}_{\rm{g}},\;i}\,+\,\frac{{h_{{\rm{t}}_{{\rm{2g}}},\;i}}}{{\frac{8}{6}}}} \right)} }},$$

where *I*_A_ is the area of feature A that is normalized both by TM content as obtained from XRF measurements (Supplementary Table 1) and by oxygen content, relative to the stoichiometric oxide, as derived from the TM oxidation state (Supplementary Table 4). *I*_A_ was determined by integration using a linear baseline between the two nearest local minima (shaded area in Fig. 2). *x*_*i*_ is the respective fractional abundance of the TM state in the catalyst and *h* the respective number of holes, both of which were determined from the oxidation and spin states in the Co L_2,3_-edge analysis. Index *i* represents different TM states abundant in the material.

**Fig. 3:**
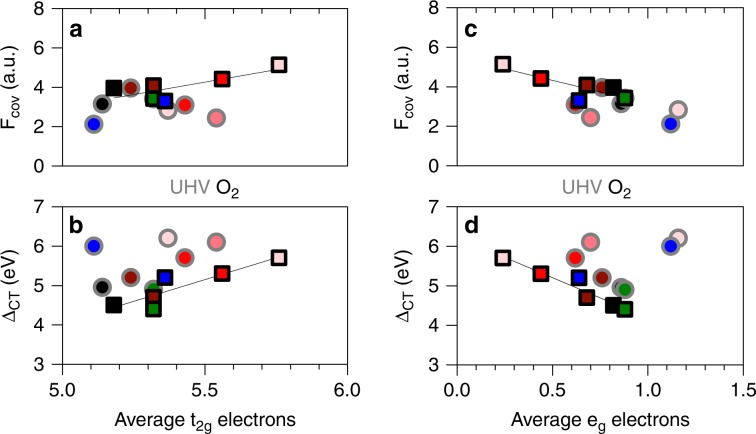
Intercorrelation of electronic parameters variation of charge-transfer energy and covalency with occupancy. Intercorrelation of electronic parameters variation of charge-transfer energy (Δ_CT_*) and covalency (*F*_cov_) with occupancy of (**a**, **b**) transition metal (TM) *t*_2g_ or (**c**, **d**) TM *e*_g_ orbitals. Symbol edge color identifies *F*_cov_ in O_2_ presence (black) or ultrahigh vacuum (UHV, gray). Fill colors represent the substituent cation: Ni (green), Zn (blue), increasing Al fraction (dark to light red), and LC (black). Lines represent least squares fits of data measured in O_2_ presence. Asterisk indicates that the charge-transfer gap was determined as the difference between energies of peak density of states for comparability with previous analysis (Supplementary Note 4). As a result true physical values are smaller than the reported values here.

Figure 3 shows electronic parameters as a function of TM 3*d*-orbital occupancy. While the results in UHV are convoluted by the varying degree of reduction among the catalysts, measurements in O_2_ presence show clearly that covalency decreases as *e*_g_ occupancy increases. When the number of unoccupied states in *e*_g_ decreases for higher abundance of HS states stronger σ-interaction of unoccupied Co^3+^ *e*_g_ states are replaced by weaker π-interactions of O 2*p* with unoccupied *t*_2g_ orbitals. This causes a reduced overall covalent binding. The covalency is slightly more pronounced in O_2_ presence compared with UHV because electron density increases through adsorption of O-species and the refilling of O vacancies that were present in UHV conditions.

The charge-transfer energy (Δ_CT_) is determined as the energy difference between O 2*p* nonbonding states in XPS valence band measurements and feature A of the O K-edge spectra^28^ (Supplementary Fig. 5). The single-valued parameter, *F*_cov_ (Eq. (1)), quantifies covalency in terms of the covalent electron density transfer from O 2*p* to TM 3*d* states. *F*_cov_ was reported to increase with the atomic number of the TM or the average oxidation state. The trends were attributed to decreasing Δ_CT_ in both cases^12^ citing lower energy levels of 3*d* states of late TM or energy shifts upon TM oxidation, respectively, for the decreasing Δ_CT_. The results at hand, however, show a concurrent increase of *F*_cov_ with Δ_CT._ This correlation is equivalent to a substantial increase of the overlap integral of O 2*p*–Co 3*d*, given the definition of covalency as ratio of overlap integral to Δ_CT_^29^. This agrees well with the conclusions above on the reasons for increasing LS state abundance and higher reducibility that originate from increasing electron density in the residual states of the Co–O substructure, particularly upon extensive Al substitution. The increased electron–electron repulsion leads to more expansive orbitals with increased overlap integral.

### Correlation of electronic parameters and reaction rates

The electronic characteristics of catalytically active sites of TM oxides are essential to the catalytic efficacy. Electronic descriptors for electro- or chemocatalytic efficacy are generally based on the analysis of either O 2*p* and/or TM 3*d* states near the Fermi-level such as 3*d*-orbital occupancy, charge-transfer energy or covalency^4,5^. The characteristics of these boundary states at active sites determine the activation of reactants through interaction with molecular orbitals. The analysis of 3*d*-orbital occupancy considers the formal oxidation and spin states of abundant TMs and thus characterizes the ionic electron density at the TM sites. This consideration neglects covalently shared electron density, which is included in the *F*_cov_ parameter. In the following the electronic characteristics at 623 K as described in the previous section are compared with surface-specific reaction rates. Charge-transfer energy will not be addressed in the following because no apparent correlation was observed, which is in contrast to the findings in electrocatalysis^4^. The surface-specific rates were measured in steady state, at ambient pressure and are corrected for deactivation, if applicable. Details are described in the “Methods” section.

### Correlations with *e*_g_ and *t*_2g_ occupancy

**Fig. 4:**
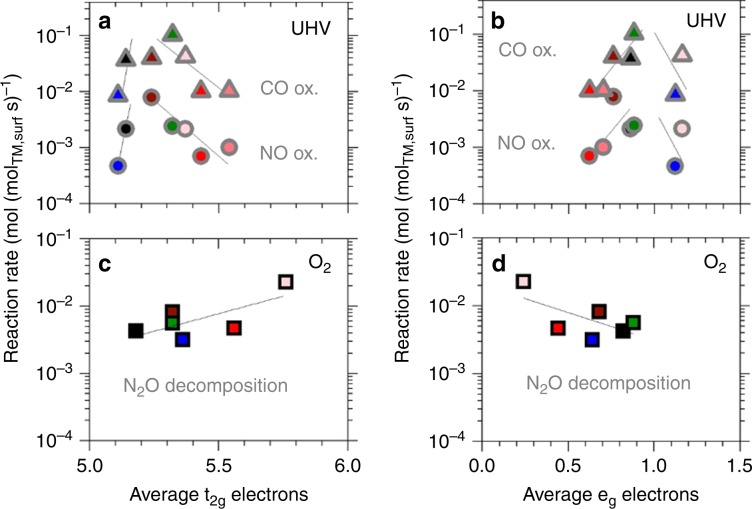
Correlations of catalytic rates and *d*-orbital occupancy. **a**, **b** Dependence of reaction rates in CO oxidation (triangles) and NO oxidation (circles) on average number of electrons in TM *t*_2g_ or *e*_g_ orbitals measured in ultrahigh vacuum (UHV). **c**, **d** N_2_O decomposition rates as a function of *t*_2g_ or *e*_g_ orbital occupancy in 0.37 kPa O_2_. Symbol edge color identifies *F*_cov_ in O_2_ presence (black) or UHV (gray). Fill colors represent the substituent cation in LaCoO_3_ (no substituent: black fill): Ni (green), Zn (blue), increasing Al fraction (dark to light red). Lines represent least squares fits of the full set or subset of data.

*e*_g_ orbital occupancy is well-known as descriptor for overpotential in electrocatalytic reactions on perovskites^3,30^ and linear or volcano-type correlations were recently reported for catalytic efficacy in oxidation of NO, CO, and hydrocarbons on various TM perovskites as well^5^. The substituent cations Al^3+^ and Zn^2+^ do not contribute as active centers and therefore do not lead to a change in *e*_g_ or *t*_2g_ orbital population directly like Ni but have an indirect effect on Co oxidation and spin state. The volcano-type correlation of e_g_ electrons with CO oxidation rates reported therein is confirmed here only in UHV XAS measurements (Fig. 4, see Supplementary Fig. 6 for correlations not shown here). A similar trend is established for *t*_2g_ occupancy. The branch of increasing NO oxidation rate with e_g_ occupancy is in contrast to previous results^5^, which was based on preexisting studies on La_*x*_MnO_3 + *δ*_^31^ and La_1 − *x*_Sr_*x*_CoO_3_^32^. The latter did not include experimental analysis of Co oxidation and spin states but instead relied on stoichiometric projections based on a BO_5_ geometry of an undercoordinated active site. Yet, the interaction with the reactant during the reaction requires the consideration of an octahedral geometry at the TM site for the determination of the 3*d-*orbital occupancy. XPS O 1*s* analysis of LCA-82 shows the presence of a superoxide species (Supplementary Note 5, Supplementary Figs. 7 and 8). Co L_2,3_-edge measurements cannot reflect an impact of varying adsorbed O-species or other relevant surface species on catalytic rates. The surface species, however, do not appear as crucial factor in the correlations in this work.

In O_2_ presence, the *d*^6^ configuration of re-oxidized Co^3+^ centers results in a complementary trend of N_2_O decomposition rates with *e*_g_ and *t*_2g_ occupancy. N_2_O decomposition rates increase for high *t*_2g_ and low *e*_g_ occupancy but the scatter in the correlation is significant. The correlation of N_2_O decomposition rates with 3*d*-orbital occupancy in UHV is markedly less directional. This disparity of correlations with reduced and oxidized state of the catalysts illustrates the relevance of measurement conditions in a correlation with individual reaction rates.

### Correlations with covalency

**Fig. 5:**
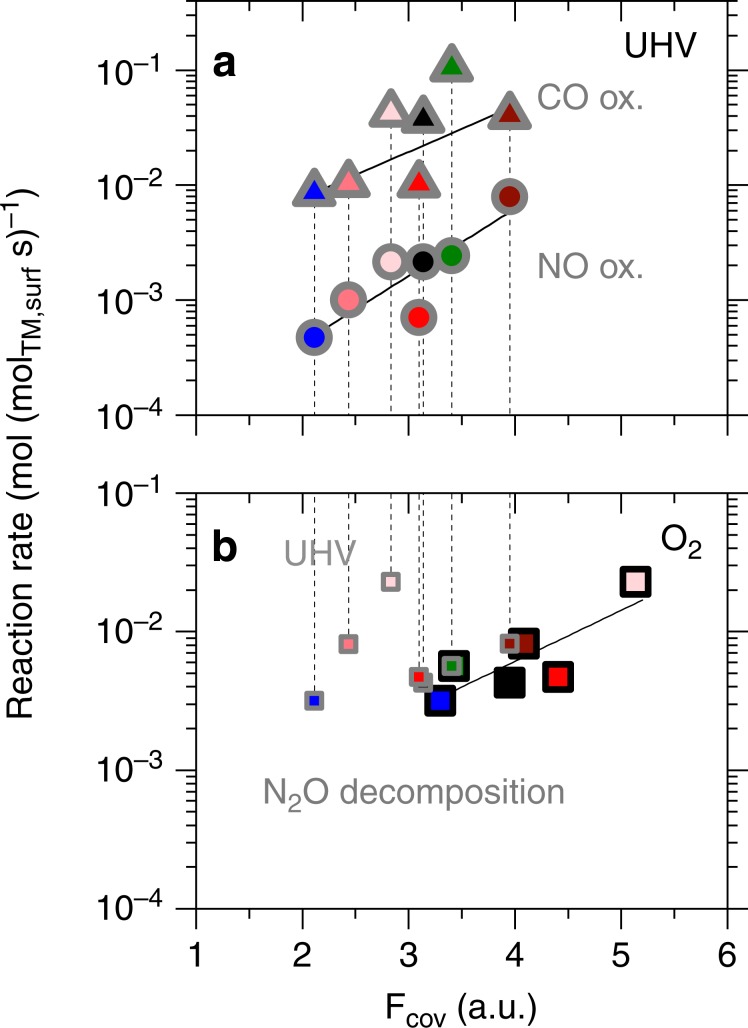
Correlation of catalytic rates with covalency. Dependence of reaction rates on covalency, *F*_cov_, at 623 K: **a** NO oxidation (523 K) and CO oxidation rates (443 K) with *F*_cov_ as determined in ultrahigh vacuum (UHV) **b** N_2_O decomposition rates (673 K) as function of *F*_cov_ in O_2_ presence (large squares) and UHV (small squares). Symbol edge color identifies *F*_cov_ in O_2_ presence (black) or ultrahigh vacuum (gray). Fill colors represent the substituent cation in LaCoO_3_ (no substituent: black fill): Ni (green), Zn (blue), increasing Al fraction (dark to light red). Solid lines represent least squares fits.

Covalency is a suitable descriptor for overpotential in electrocatalytic OER and ORR on TM perovskites^3,12^, but has not been related to surface-specific reaction rates of chemocatalyzed reactions. Here, we compare the reaction rate per active TM site with *F*_cov_, which represents the electron density transfer through covalent interaction of O 2*p* and TM 3*d* states that is affected by the non-TM substituent cations as described above. Both NO and CO oxidation rate, the latter less accurately, are described by covalency measured in UHV, but not in O_2_ presence (Fig. 5). N_2_O decomposition rates, in contrast, correspond with covalency in O_2_ presence only. We conclude from these results that the covalently shared electron density at TM 3*d* states (*F*_cov_) is a decisive characteristic in catalytic reactions when adequate measurement conditions are applied. Increased covalency in the oxide implies the ability of a catalyst active site to form a more pronounced covalent interaction with frontier molecular orbitals of surface species through a larger overlap integral and thus facilitates electron density transfer in redox reactions. The correlation with the overall reaction rates implies that the ability of covalent interaction with the reactant lowers the activation energy in the kinetically relevant step of the reaction, which occur through one or the combination of an energetically stabilized transition state and elevated energy of reactants in the kinetically relevant step. These considerations assume that effects in confined spaces or of adjacent surface species do not occur or do not vary significantly among the catalysts in the reaction systems studied here. The dependence of covalency as descriptor of reaction rates on experimental conditions that is similar to the correlations with *e*_g_ or *t*_2g_ occupancy described above, however, is crucial and is addressed in the following.

The crucial role of measurement conditions implies that a reaction-specific catalyst state has to be recreated in XAS measurements to acquire relevant information for descriptor correlations. The relevant conditions are closely related to individual reaction characteristics such as most abundant surface intermediates and the kinetically relevant step of a catalytic reaction. These characteristics are available through kinetic analysis of the reaction mechanism that allows to portray the catalyst state during its interaction with frontier orbitals of reactants and the transition state in the kinetically relevant step. Thus, valid correlations are tied to a specific XAS measurement condition, here UHV or O_2_.

**Fig. 6:**
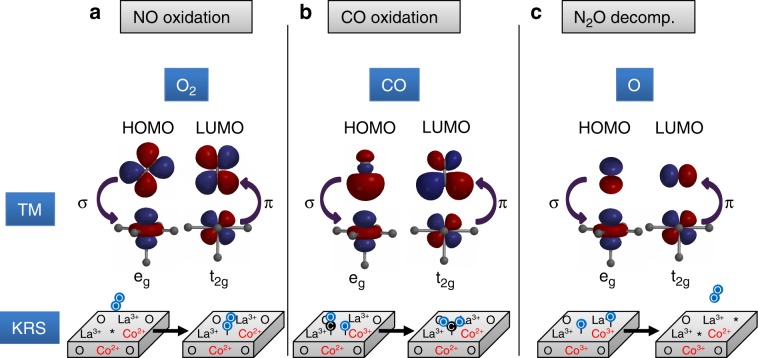
Interaction of orbitals of the active site and surface species. Reactant-active-site interactions involve σ- and π-interactions of the transition metal (TM) 3*d* orbitals with the highest occupied (HOMO) and the lowest unoccupied molecular orbital (LUMO) of the reactant. Orbital interactions and the respective kinetically relevant step (KRS) are shown for (**a**) NO oxidation, (**b**) CO oxidation, and (**c**) N_2_O decomposition. Please note that the depiction of the KRS does not include the complexity of binding sites on the catalyst, particularly for CO oxidation.

Mechanistic analysis of NO oxidation on Co-based  TM oxides concluded that O* is the sole most abundant surface intermediate and O_2_ adsorption on dilute vacant sites is the kinetically relevant step^22^. The O_2_ adsorption, which occurs in linear or angled geometry^33^ and enables σ- and π-interaction with *e*_g_ and *t*_2g_ orbitals, respectively (Fig. 6), is redox-neutral and does not oxidize the previously undercoordinated active site. Therefore, NO oxidation rates only correlate with covalency in UHV measurements which capture the characteristics of partially reduced TM sites. XAS measurements in O_2_ presence, in contrast, characterize a fully oxidized catalyst and thus do not provide the relevant information on electron density at the active site during the adsorption of O_2_.

A similar correlation is present for covalency in UHV and CO oxidation rates, despite a significantly different reaction mechanism for CO oxidation compared with NO oxidation: the surface reaction of adsorbed CO* and O* is the kinetically relevant step on LaCoO_3_ and separate adsorption sites were determined for O* as well as CO* and CO_2_^*^^34^. The dependence of CO oxidation rates on reactant partial pressure on LC was confirmed in this study (Supplementary Note 6, Supplementary Fig. 9). The two-site mechanism is in line with the previous report that both TM and O states affect catalysis on TM perovskites^9^ and further underlines the relevance of covalency as it affects both TM and O sites. Akin to NO oxidation, the correlation with covalency in UHV implies that the catalyst is in a partially reduced state during the kinetically relevant step, which is due to the nonoxidative and π-acceptor nature of CO. The comparably high scatter in the correlation of CO oxidation rates with *F*_cov,UHV_ is ascribed to the more complex multisite reaction step together with measurements in absence of CO. The complexity merits an in-detail XAS investigation in a reaction mixture of CO, O_2_, and CO_2_.

N_2_O decomposition rates correlate with increasing covalency in O_2_ presence, while reducible materials are outliers in the respective correlation in UHV (Fig. 5). Covalency as descriptor relates to the characteristics of the kinetically relevant step of associative desorption of O anions as O_2_, which has been determined from the inhibition effect of O_2_ and correlations with surface oxygen exchange rates on perovskite-type TM oxides.^35–37^ The interaction with the adsorbed O anion and the electron density transfer in the oxidative reaction to O_2_ therefore defines the catalytic efficacy (Fig. 6): the active site is oxidized at the start of the kinetically relevant step and partially reduced after the desorption of O_2_. The correlation of the overall rate of N_2_O decomposition only with covalency in O_2_ presence implies that the oxidized state of the catalyst is relevant in the kinetically relevant step. This finding hints at an early transition state in the associative desorption of O_2_ because the active site is still fully oxidized before the kinetically relevant step but is left partially reduced after the desorption. The contrast to NO oxidation, where the nonoxidizing molecular adsorption of O_2_ is the kinetically relevant step, is therefore caused by the different characteristics of the respective kinetically relevant step despite the similar surface species involved.

## Discussion

Correlation of 3*d*-orbital occupancy with NO and CO oxidation rates results in volcano-type plots, that we find partially in dissent with previous reports on NO oxidation^5^. The dissent emphasizes the need to employ conditions that are similar to the reaction parameters for the analysis of Co or TM states. While the occupancy of *d*-orbital states is a valid descriptor, it is generally unable to represent the full picture of the relevant electronic characteristics of a catalyst as it only accounts for formal TM spin and oxidation states. In contrast, the single-valued covalency factor, *F*_cov_, is able to encompass the effects of covalent TM–O interaction. The correlations in the present study show *F*_cov_ as a viable descriptor of surface-specific reaction rates in three reactions: NO oxidation, CO oxidation, and N_2_O decomposition. The results indicate TM–O covalency as a critical parameter for catalytic rates on Co-based perovskite-type oxides and maximizing covalency as target for intrinsic catalyst design.

The reported correlations are strongly dependent on XAS measurement conditions, particularly in terms of oxidizing or reducing conditions. This effect is attributed to the different characteristics of the surface and catalyst–reactant interaction in the transition state in the respective kinetically relevant step. This association between measurement conditions during XAS and information from kinetic analysis underpins the requirement of mechanistic understanding. The XAS measurements in UHV or in O_2_ presence in this study are a simplification of the respective reaction systems. The impact of measurement conditions and surface species merits additional measurements in presence of a more characteristic reactant mixture to elaborate on the details of their effects. In addition, a broader analysis of perovskite-type oxides with different TM cations is in line to determine whether covalency is a viable concept to compare catalytic rates across perovskite-type oxides with different compositions which has been shown for electrocatalysis^3,12^. The analysis of covalency in perovskites is state of the art for different TM cations^12^.

The conclusion that covalency is a crucial parameter for catalytic rates on perovskite-type oxides is an interesting concept for other TM oxides as well. The comparison of different crystal structures is potentially more complex, but covalent TM–O interactions are well-described for many TM oxides^38^. The electronic characteristics of TM oxides are primarily based on the interactions of TM cations and O^2−^ in the catalyst. Thus, a further improved understanding of the variation of covalency, its relation to reaction mechanisms and catalytic rates will elaborate an important aspect in catalysis on TM oxides. In addition, a brief exemplary analysis of catalytic N_2_O decomposition illustrates that varying electronic characteristics have been employed as descriptors for this reaction on TM oxides with different crystal structures. N_2_O decomposition efficacy on CoOx/ZrO_2_ increases with electron density in the CoOx clusters^39^ which represents the same trend as we show here for the covalency in perovskite-type materials. Another reported descriptor for catalytic efficacy in N_2_O decomposition is the catalyst work function^40–42^. The work function in TM oxides is also dependent on *d*-band filling^43,44^ as it describes the energy required to remove an electron from states near the Fermi-level that are dominated by O 2*p* and TM 3*d* contribution. Thus, both covalency and the work function are based on electronic characteristics of the same boundary states. Another structural parallel emerges from a comparison of the materials with varying Al substitution degree in the current work with Co-containing hexaaluminates, which exhibit the highest N_2_O decomposition rates among TM oxides that contain octahedrally coordinated Co^3+^ cations^16^. The very low Co/Al ratio in hexaaluminates constitutes an extrapolation of the LaCo_1 − *x*_Al_*x*_O_3_ system and would be in line with a continued increase in covalency along with substitution degree (Fig. 5) that results in higher catalytic efficacy compared with other Co-containing oxides. These exemplary parallels in the electronic characteristics indicate strongly that catalytic descriptors may be similar across different crystal structures. The presence of different approaches merits further experiments to explore the correlations among different electronic parameters themselves and their capability to predict catalytic efficacy.

While this work presents evidence of covalency as highly relevant descriptor for Co-based perovskite-type oxides, a broader use depends on its applicability to other TM cations. In this work, mechanistic conclusions are assumed valid for all materials tested, which is underpinned by the resulting correlations. A varying nature of the TM cation may, however, affect active-site interaction with the different reactants strongly and cause a different kinetic regime. More complex redox-characteristics, Mn^3+/4+^ and Co^2+/3+^ are present in LaCo_1 − *x*_Mn_*x*_O_3_ for example^14^, may further complicate the interactions and respective reaction mechanism as Mn is catalytically active as well and the oxidation states of the two TM sites become relevant. This possible variability of the kinetically relevant step may then require different XAS experimental conditions for the same reaction on different catalysts to recreate the appropriate catalyst state for descriptor correlations. This work, which focuses on the complex effects of substitution in Co-based perovskite-type, therefore marks a starting point to the further analysis and potentially broader application of covalency as descriptor for the catalytic efficacy of TM oxides.

## Methods

### Synthesis and phase analysis

Single-phase perovskite-type oxides were prepared via a citrate route, similar to a method reported in literature^2^. Precursor compounds (Ni(NO_3_)_2_·6H_2_O (Carl Roth, 99%), Co(NO_3_)_2_·6H_2_O (Carl Roth, >98%), La(NO_3_)_3_·6H_2_O (Carl Roth, 99.99%), Al(NO_3_)_3_·9H_2_O (Carl Roth, >98%), Zn(NO_3_)_2_·6H_2_O (Carl Roth, >99%), and CA (Carl Roth, 99.5%, water free)) were used without further purification. The metal precursors in the desired molar ratio were dissolved in 100 cm^3^ deionized water together with CA, which was added in 10% molar excess (CA:sum of cations = 1.1:1) for complete complexation of the metal cations. The solution was stirred for 1 h at 353 K prior to water removal in a rotary evaporator at 323 K and 5.0 kPa total pressure for 2 h and the resulting viscous slurry was dried overnight at 393 K in stagnant air. The obtained sponge-like solid was crushed and treated in stagnant air for 2 h at 573 K (0.033 K s^−1^) and for 8 h at 973 K (0.066 K s^−1^) in flowing dry air (1.7 cm^3^ s^−1^, Westfalen, 99.999%). After cooling down the obtained perovskite material was used for analysis and catalytic measurements.

X-ray diffractograms were obtained using a Cu Kα source and a Bragg–Brentano geometry in a Siemens D5000 powder diffractometer. The data was acquired in a range of 2θ = 3–90° with a step width of 0.02°. Lattice parameters were determined by profile fitting (FullProf^45^). N_2_-physisorption was used to determine the surface area of LaCoO_3_ using the multipoint BET method^46^ from isotherms recorded on a Quadrasorb SI by Quantachrome Instruments after degassing of materials (0.4 kPa total pressure at 393 K for minimum 4 h)

X-ray fluorescence, XRF, measurements were performed with a micro X-ray fluorescence spectrometer (EAGLE μ-Probe II, (Oxford Instruments, Abingdon, GB-OXF)) with a 300 μm capillary and operated at 40 kV using a Rh target.

### In situ XAS and XPS

Ambient pressure XAS and XPS was measured at beamline 11.0.2^47^, Advanced Light Source (Lawrence Berkeley National Laboratory). All data were collected in a single beamtime under multi-bunch operation. A custom-made multisample holder (304 stainless steel) was used for delivery of four samples at once into the UHV chamber. Nonporous pellets for use in UHV were obtained from powder samples of the catalyst by cold isostatic pressing at 690 MPa and room temperature. Part of the obtained catalyst pellet was pressed into a cavity of the sample holder using a custom-made die (304 stainless steel) to result in a level surface of the catalyst. A ceramic heater with Pt–Ir electrical probes in combination with an YSZ ionic resistance, which was calibrated inside the blackbody environment of a tube furnace, was used for temperature control. The beamline features a SPECS Phoibos 150-DLD 1D electron energy analyzer and a slitless variable-included-angle plane grate monochromator.

The catalysts were pretreated at 673 K in UHV for 30 min. The experimental conditions were varied in temperature and in UHV or oxidative atmosphere (0.37 kPa O_2_), XAS and XPS measurements were first taken in UHV in a temperature sequence of 623 K→523 K→423 K, and consecutively in O_2_ presence (0.37 kPa), at 423 K and then 623 K. An equilibration period of 10 min was used when experimental parameters were altered. Each catalyst in the sample holder was measured at a particular set of experimental conditions before new conditions were applied. The first sample holder contained LC, LCA-82, LCA-64, and LCA-46, while the second set of catalysts included LC, LCA-28, LCZ-82, and LCN-82. Some materials were measured only at select conditions and all spectra were normalized by their respective postedge intensity as described below. The beam spot size was 200 × 60 μm or 200 × 20 µm for XPS or XAS, respectively. The beam spot was altered for one catalyst after each change of experimental conditions. Beam influence was further monitored by comparing repetitive measurements of Co 2*p* and O 1*s* XP spectra at the beginning and the end of the set of measurements.

XAS was acquired in partial-electron-yield detection mode. The inelastic mean free path of electrons in the samples was calculated to be ~0.6 nm using the QUASES-IMFP-TPP2M software package^48^. Interference with Auger and photoelectron peaks was circumvented by choice of the kinetic energy window of the detected electrons at 305 eV ± 10 eV for Co, and 295 eV ± 10 eV for O and Ni with a pass energy of 100 eV. Each O K-edge X-ray absorption spectrum is normalized by its postedge intensity at 548–550 eV. Subsequently, a linear background was subtracted that was determined by averaged intensity before (523–526 eV) and after the edge (548–550 eV). Data for Co L_2,3_-edges were processed analogously at corresponding energies before (770–773 eV) and after the edge (805–810 eV).

Spectral contribution of gaseous oxygen species (530.4 eV and 536/540 eV^49^) in O K-edge measurements was accounted for by background subtraction. The background spectrum^9^ of isolated gaseous O_2_ absorption was used, which was taken as well at ALS beamline 11.0.2.

The core-level spectra (O 1*s*, C 1*s*, La 4*d*, Al 2*p*, and Co 3*p*) of LCA-64 were measured in UHV (∼10^−7^ Torr) at ambient temperature. The C 1*s* binding energy of adventitious carbon at 284.8 eV was used as reference for the spectra^50^. After the pretreatment carbon species remained on the surface as apparent in C 1*s* peaks at elevated binding energy compared with adventitious carbon, some of which hydrofluorocarbon as F 1*s* peaks appeared in survey measurements for some samples. Subsequent XPS measurements (C 1*s*, O 1*s*, La 4*d*, Co 3*p*, valence band as well as Ni 3*p*, Zn 3*p*, Al 2*p*, where applicable) were thus referenced to the bulk species in O 1*s* and included differential energy measurements to assess depth-dependent variation using kinetic energies from 200 to 465 eV for O 1*s* species.

The analysis of different surface species on the perovskite surface in O 1*s* spectra was conducted using Gaussian–Lorentzian peaks and a Shirley-type background subtraction to deconvolute different species. The fit of the O 1*s* region at 735 eV assumed contributions of bulk O^2−^ (“bulk”), hydroxyls OH, (bi)-carbonates, negatively charged “surface” oxygen, and, where applicable, weakly charged oxygen species. The main interest is the presence of the weakly charged oxygen species. Analysis is therefore constrained to binding energy considerations and neither detailed peak fitting parameters nor an analysis of the fraction of carbonate species is included in this work. The fitting systematic is equivalent to previous analyses of surface species on Co-based perovskites^51^ but was done with a constant line shape with 70% Gaussian and 30% Lorentzian character for all species.

### Reaction rate measurements and calculation

The catalytic material together with quartz as inert diluent was pressed to 200–355-µm-diameter particles in a U-shape quartz glass reactor with a diameter of 0.5 cm and a total mass of diluent and catalyst of 0.060 g or with a diameter of 0.81 cm and a total mass of 0.200 g. The calculation of the number of surface sites is based on the number of B-site cations per 001 facet of the ideal cubic perovskite structure, which is the most stabile facet^52^. Lattice structure and lattice parameters of LaCoO_3_ were considered to derive a value of 7 Co^3+^ atoms nm^−2^ for the 001 facet of the perovskite structure. The combination with BET surface area and the fraction of TM cations on the B-site as obtained from XRF allows to calculate the number of TM cations on the surface. Zn, with a closed *d*^10^ configuration is not included in the number of TM cations because it is not considered as active center as its electronic states are well below the Fermi-level.

The catalyst was pretreated in He or N_2_ flow for 0.5 h at 673 K prior to NO oxidation or 873 K prior to N_2_O decomposition and CO oxidation, and subsequently cooled down to the reaction temperature. Reactant flow was initiated upon reaching the desired reaction temperature after pretreatment. For NO oxidation, NO and NO_2_ gas concentrations were monitored using an IR gas analyzer (MKS, Multigas Analyzer Model 2030) equipped with a 2 cm gas cell held at 353 K. N_2_O and CO concentrations were continuously monitored via a quadrupole mass spectrometer (m/z = 44, MKS Instruments, Cirrus 2) equipped with a faraday cup and electron multiplier detector, or via FT-IR (Perkin-Elmer, Spectrum Two) equipped with a 500 mL gas cell (Pike Technologies Inc), respectively. Overall, 10% of He was used as internal standard in the MS analysis.

For reaction rate data, a K-type thermocouple was attached to the glass reactor to control and monitor the temperature at the catalyst bed using a resistively heated furnace. The flow was held constant at 3 cm^3^ s^−1^ using He or Ar as balance gas. Gas concentrations were controlled with parallel mass flow controllers and held at 0.10 kPa NO, 0.03 kPa NO_2_, and 5.15 kPa O_2_. All gases were obtained from Praxair. N_2_O decomposition was tested with 0.10 kPa N_2_O and 5.15 kPa O_2_, while inlet concentrations for CO oxidation were 0.20 kPa CO and 5.15 kPa O_2_. Information on the gases used is presented in Supplementary Table 5.

Differential changes in NO, CO, or N_2_O concentration along the catalytic bed were approximated by use of the mean of reactor bed inlet and outlet concentration, which was derived from the conversion, for the calculation of reaction rates. Differential changes in NO_2_ were approximated via the inverse mean concentration as shown in Eq. (2) to account for the inverse dependence in the rate equation^53^.

2 $$P_{\rm{NO2,mean}}\,=\,\frac{1}{{\frac{{\frac{1}{{P_{\rm{NO2,in}}}}\,+\,\frac{1}{P_{\rm{NO2,out}}}}}{2}}}\,=\,\frac{2}{{\frac{1}{P_{\rm{NO2,in}}}\,+\,\frac{1}{P_{\rm{NO2,out}}}}}.$$

Forward NO oxidation rates were calculated from experimental data by correcting for the approach to equilibrium by use of Eqs. (3) and (4)^54^.

3 $$\eta\,=\,\frac{{\overrightarrow r_{\rm{NO}}}}{{\begin{array}{*{20}{c}} {{{\overleftarrow r_{\rm{NO}}}}} \end{array}}}\,=\,\left( {\frac{{P_{\rm{NO}}^2P_{\rm{O2}}}}{{P_{\rm{NO}}^2K_{\rm{eq}}}}} \right),$$

4 $$\overrightarrow r_{NO}\,=\,\frac{{r_{NO,{\mathrm{exp}}}}}{{1\,-\,\eta }}.$$

The values for the thermodynamic equilibrium constant *K*_eq_ were calculated from tabulated data^55^. In addition, the rates were corrected for contribution of the gas phase reaction of NO and O_2_ by empty reactor blind tests. Steady state reaction rates were interpolated to the given temperature and are normalized to the TM (Co + Ni) content as presented in Supplementary Table 1. Deactivation was linearly corrected for by comparing rate measurements at the same experimental conditions at the start and the end of a set of measurements.

## Supplementary information

Supplementary Information

## Data Availability

Data are available from the authors upon reasonable request.

## Code availability

All information on used code or mathematical algorithms is available from the authors upon reasonable request.
