# Supplementary material for: Electronic parameters in cobalt-based perovskite-type oxides as descriptors for chemocatalytic reactions
Source: Nat Commun. 2020 Jan 31;11:652. doi: 10.1038/s41467-020-14305-0 (PMC6994687; doi:10.1038/s41467-020-14305-0)
Supplement: Supplementary file 1 — Supplementary Information [file 41467_2020_14305_MOESM1_ESM.pdf]

Supporting Information

Electronic parameters in cobalt-based perovskite-type oxides as descriptors for chemocatalytic reactions

Simböck et al.

Supplementary Note 1: Results of X-ray diffraction analysis

The diffractograms (Supplementary Figure 1) of the catalysts show that phase-pure rhombohedrally distorted $LaCoO_3$, space group R-3c, (ICDD # 00-009-0358) was obtained for unsubstituted and Ni-substituted material. Zn and Al substituted catalysts crystallize in the ideal cubic perovskite structure, space group Pm-3m. Al substitution yielded similar results before [1]. A closer look at the largest reflex at 2θ = 33° illustrates that the size of the substituent cation relative to Co^{3+} causes a shift to lower (Zn, Ni) or higher degrees of 2θ (Al), which is also reflected in the cell parameters shift.

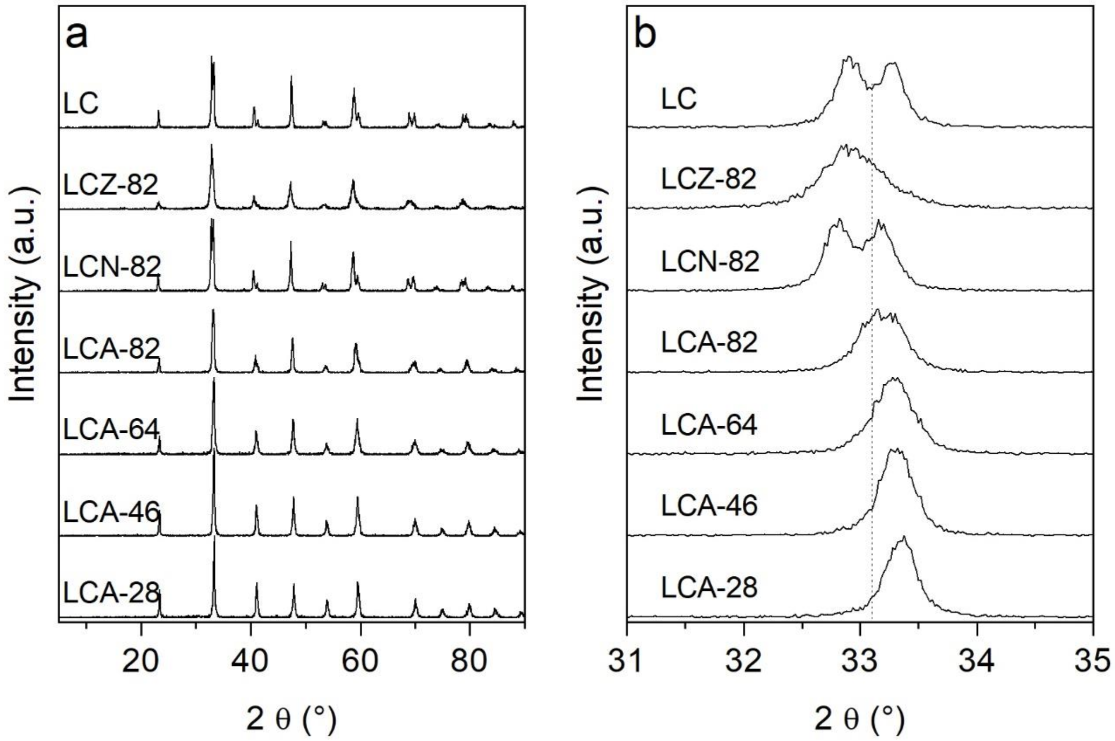

Supplementary Fig. 1 X-Ray diffractograms of catalysts. **a** Full scale of diffractograms. **b** Enlarged main reflex.The dotted line indicates the center of the LC double reflex.

Supplementary Table 1 Lattice parameters and TM content at B-site of the perovskite.

Catalyst	Space group	Cell parameters* / Å or °					Fractional TM content on B-site	
		a = b	c	α	β	γ	Per XRF	nominal
LC	R -3 c	5.443	13.125	90	90	120	0.98	1
LCZ-82	P m -3 m	3.846	3.846	90	90	90	0.81	0.8
LCN-82	R -3 c	5.456	13.092	90	90	120	1.04	1
LCA-82	R -3 c	5.401	13.093	90	90	120	0.78	0.8
LCA-64	P m -3 m	3.804	3.804	90	90	90	0.56	0.6
LCA-46	P m -3 m	3.798	3.798	90	90	90	0.36	0.4
LCA-28	P m -3 m	3.795	3.795	90	90	90	0.21	0.2

*Determined in profile fitting of diffractograms in diffractograms (Supplementary Figure 1)

Supplementary Note 2: Analysis of spin and oxidation states

XAS at first row transition metal (TM) $L_{2,3}$-edges measures excitation of 2p electrons into unoccupied 3d states. It thus probes the energetic level and density of states of the latter, which depend on spin state, valence and coordination geometry. Supplementary Figure 2a shows the occupancy of d orbitals for the abundant Co spin and oxidation states in the octahedral geometry. The determination of Co oxidation states required to account for different spin state contributions in Co^{3+} (low spin, LS, or high spin, HS) by appropriate choice of reference spectra[2]. Subsequently, present Co^{2+} (stable HS) contribution was subtracted from the experimental data before the spin states of Co^{3+} were assessed with suitable reference spectra[3]. Quantification for all states was conducted via least squares fit of a linear combination of references (Supplementary Figure 2b) to experimental data followed by integration of the respective curve areas. Very low abundance of Co^{3+} high spin states or Co^{2+} was not accessible in the quantification and is designated as < 5 % in the respective measurements, reflecting an estimated determination threshold for low abundance.

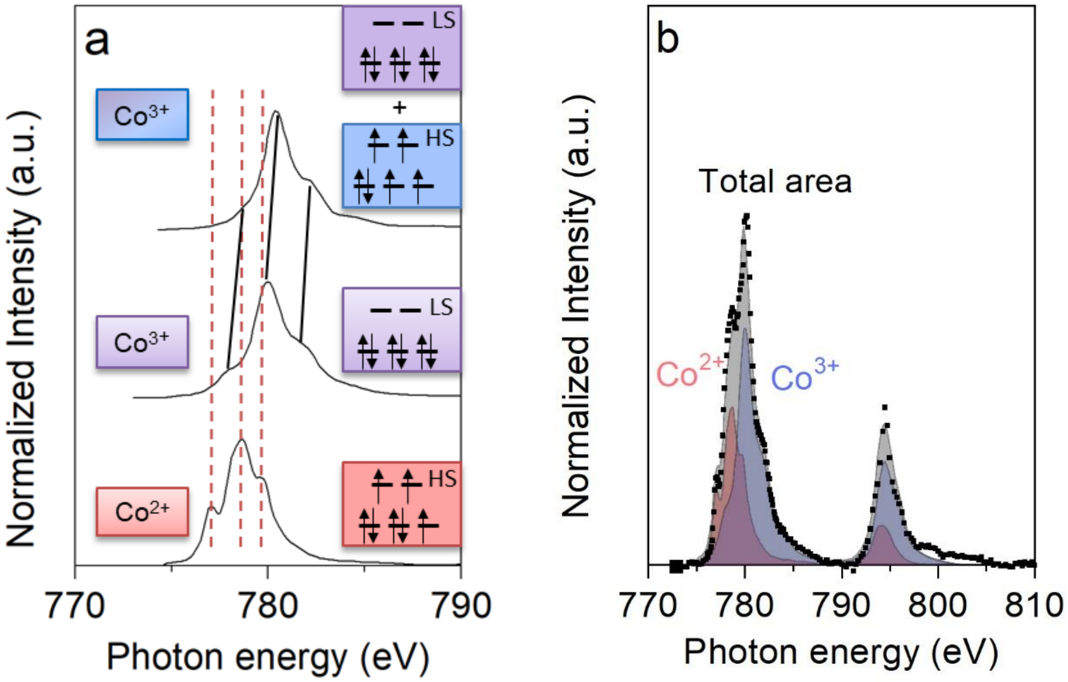

Supplementary Fig. 2 Analysis of Co oxidation and spin states. **a** Co states present in the catalyst and the respective simulated spectra of LS Co^{2+} and Co^{3+} (LS) in octahedral coordination from ref.[2] as well as mixed Co^{2+} spin states from ref.[3]. **b** Co^{3+} Analysis of TM states, here for LCA-28 measured in UHV at 623 K. Gray area represents the total area of Co^{2+} and Co^{3+}, while the scatter plot shows experimental data.

Supplementary Table 2 HS population of Co^{3+} cations at different conditions in percent.

	UHV			0. 37 kPa O_2	
	423 K	523 K	623 K	423 K	623 K
LC	32	42	43	29	41
LCZ-82	33	n.a	43	n.a.	32
LCN-82	n.a	n.a	43	n.a.	42
LCA-82	27	32	38	19	34
LCA-64	11	22	27	7	22
LCA-46	6	12	15	n.a.	n.a.
LCA-28	<5	n.a	11	<5	12

Supplementary Table 3 Abundance of Co^{2+} in the catalyst at different conditions.

	UHV			0.37 kPa O_2	
	423 K	523 K	623 K	423 K	623 K
LC	< 5	< 5	< 5	0	0
LCZ-82	14	n.a	25	n.a	0
LCN-82	n.a	n.a	< 5	n.a	0
LCA-82	< 5	< 5	< 5	0	0
LCA-64	< 5	< 5	< 5	0	0
LCA-46	25	27	24	n.a	n.a
LCA-28	52	n.a	53	0	0

Supplementary Table 4 Oxygen content in the perovskite-type oxides at different conditions.

	UHV			0. 37 kPa O_2	
	423 K	523 K	623 K	423 K	623 K
LC	2.98 -3.00	2.98 -3.00	2.98 -3.00	3.00	3.00
LCZ-82	2.85	n.a	2.80	n.a	2.90
LCN-82	n.a	n.a	2.98 -3.00	n.a	3.00
LCA-82	2.98 -3.00	2.98 -3.00	2.98 -3.00	3.00	3.00
LCA-64	2.98 -3.00	2.98 -3.00	2.98 -3.00	3.00	3.00
LCA-46	2.96	2.95	2.96	n.a	n.a
LCA-28	2.95	n.a	2.94	3.00	3.00

Values calculated based on charge neutrality and Co^{2+} abundance and Zn^{2+} content.

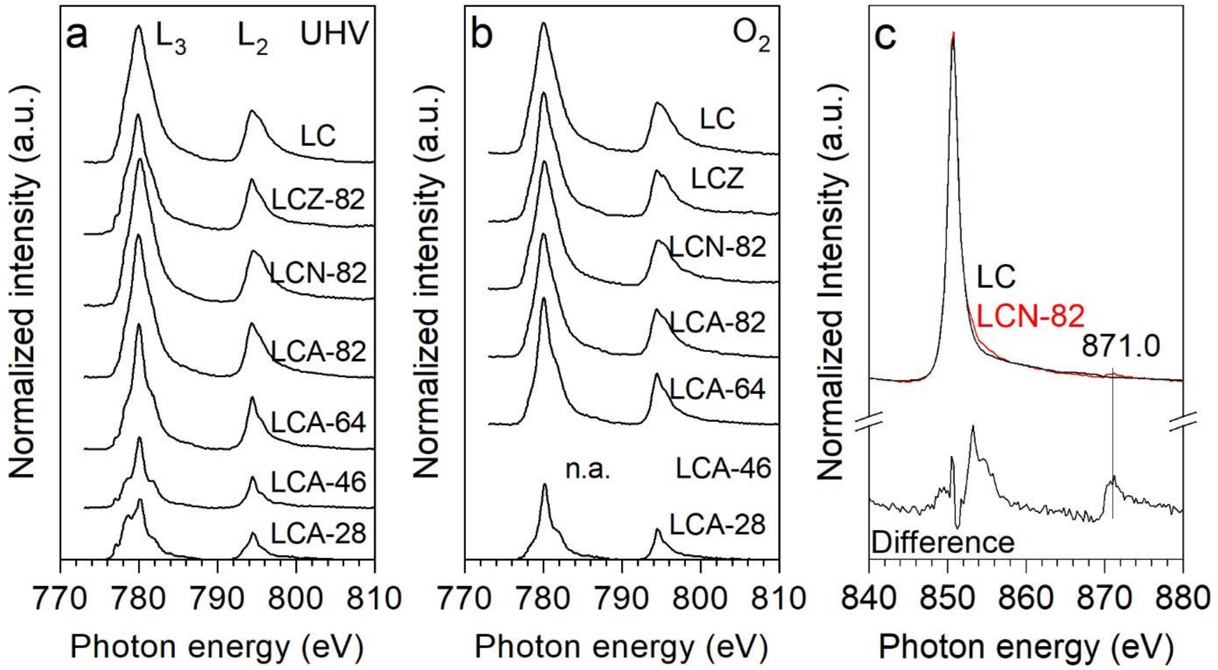

Supplementary Fig. 3 Co $L_{2,3}$-edges of all catalysts and the Ni $L_{2,3}$-edge of LCN-82. Data acquired at 623 K **a** in UHV and **b** in 0.37 kPa O_2. **c** Ni $L_{2,3}$-edges at 423 K in UHV compared to LC and the respective difference spectrum. L_2-edge peak at ~871 eV reflects Ni^{3+} in LS spin[4].

Supplementary Note 3: O K-edge analysis

The excitation of O 1s core electrons into O 2p states in O K-edge XAS quantifies O 2p states that are unoccupied because of hybridization of O 2p with cationic states. The three distinct features A, B and C in the O K-edge of LC (Supplementary Figure 4) are dominated by O 2p-TM 3d hybridized states (~ 527-531 eV), O 2p-La 5d (~ 532-538 eV) and a mixture of O 2p-La 5d and O 2p-TM 4sp states (~ 539-548 eV), respectively[5]. The O K-edge of LC at 623 K in UHV is similar to previous literature reports[6], but changes significantly with substitution. Intensity of feature A decreases with substitution of Co^{3+} by Al^{3+} because the latter lacks relevant 3d states, while peak intensity of feature B increases due to the occurrence of Al 3p - O 2p hybridized states in this energy range[7].

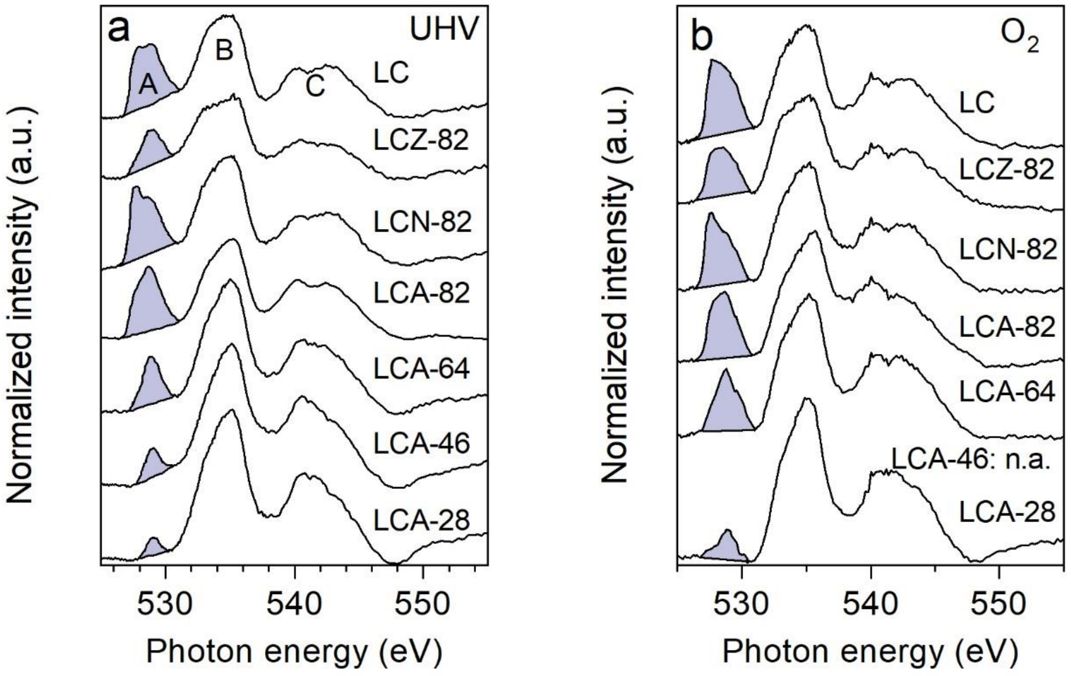

Supplementary Fig. 4 Analysis of O K-edge spectra. O K-edge of catalysts at 623 K **a** in UHV and **b** in 0.37 kPa O_2.

Supplementary Note 4: Determination of the charge-transfer energy Δ_{CT}

The energies of XPS and XAS O K-edge data were aligned on a common energy scale by a rigid shift of XAS energies. The rigid shift parameter was chosen to match results for the charge-transfer energy Δ_{CT} of for $LaCoO_3$ in[8], where alignment was established, described and analyzed in detail using complementary DFT calculations and XES measurements that are not available in this work. The rigid shift leads to a systematic change in values of Δ_{CT} and therefore trends among the oxides in this work remain valid regardless of the absolute value of a rigid shift parameter. This approach approximates that final state effects are similar among the oxides studied in this work.

It has been established that XPS valence band peaks agree well with element specific XES peaks [8]. Thus, the position of peaks in valence band spectra acquired via XPS can be used to determine electronic parameters such as the charge-transfer energy Δ_{CT} values. LCZ-82 and LCA-28 did not show well-discernible peak maxima for the O 2p band in the valence band while other catalysts show a distinguishable double-peak feature that is reported for LC [8]. Therefore, the position of the peak that refers to the non-bonding O feature was estimated to be at one third of the total breadth of the O 2p band. Δ_{CT} values were determined as the energy difference of the peak center of the O K-edge feature that relates to O 2p-TM 3d interaction and the peak of the non-bonding O states in the XPS valence band measurement to be comparable to literature data cited above.

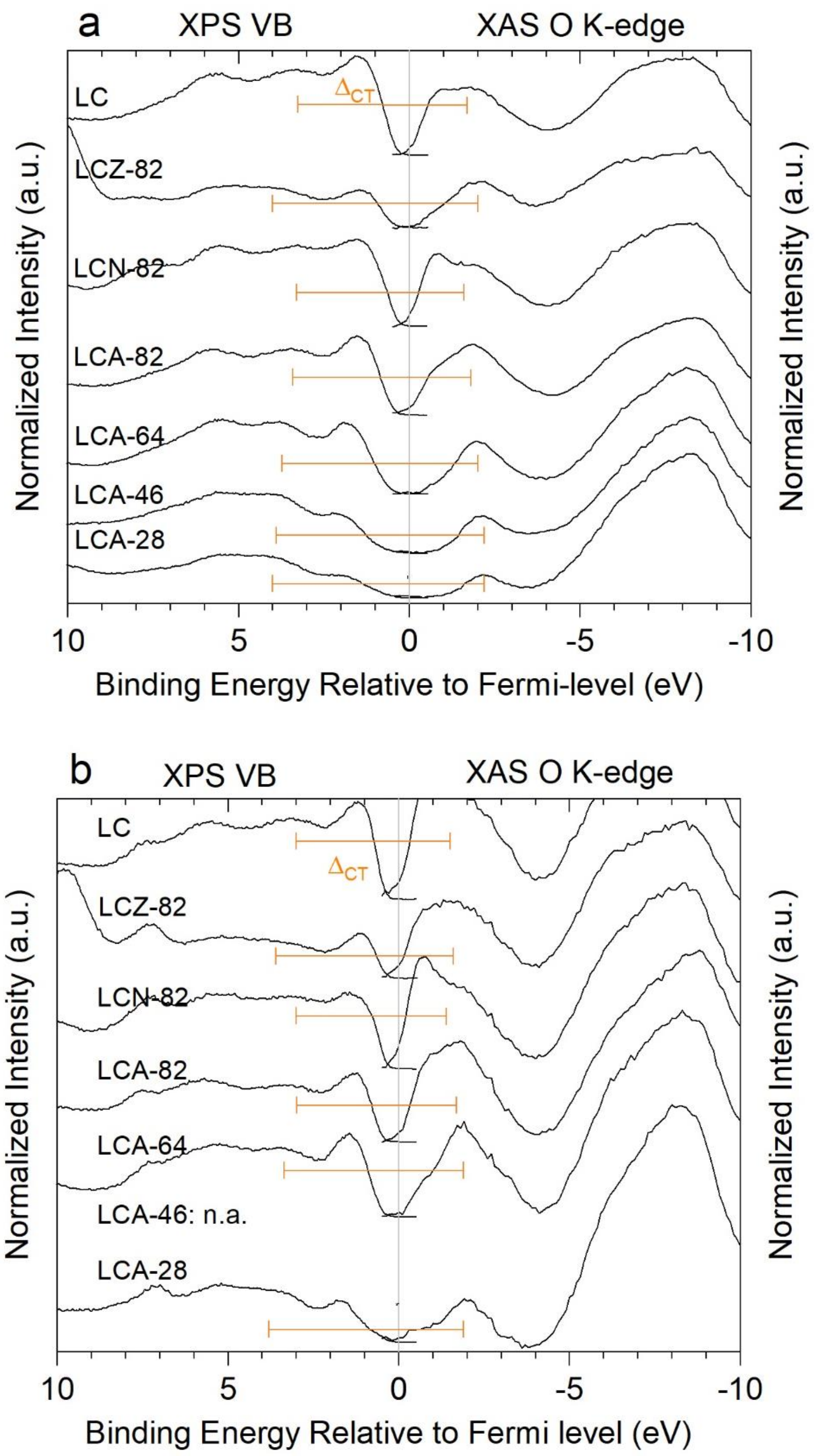

Supplementary Fig. 5 Determination of charge-transfer energy. Determination of charge-transfer energy (Δ_{CT}) at 623 K **a** in ultra-high vacuum (UHV) and **b** in 0.37 kPa O_2. XPS valence band data and XAS O K-edge data were normalized separately in arbitrary numbers. Absolute intensities are therefore not comparable.

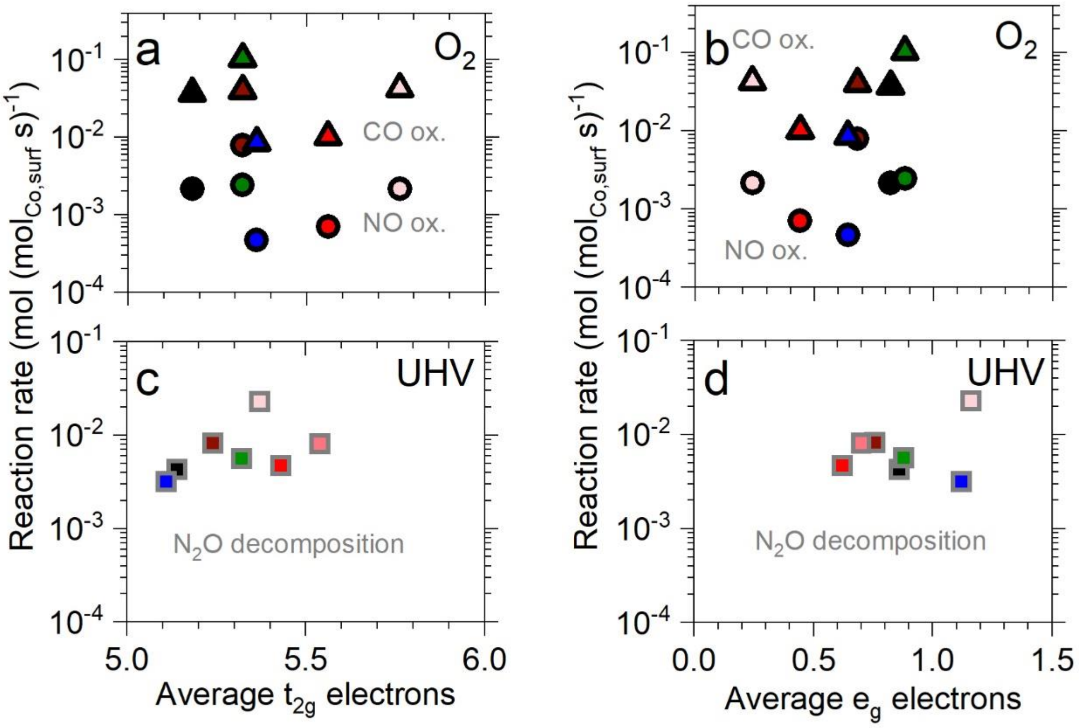

Supplementary Fig. 6 Additional plots regarding dependence of catalytic rates on d orbital occupancy. **a, c** Dependence of reaction rates in CO oxidation (triangles) and NO oxidation (circles) on average number of electrons in TM d orbitals measured in O_2 presence. **b, d**: N_2O decomposition rates as a function of 3d orbital occupancy in UHV. Symbol edge color identifies F_{cov} in O_2 presence (black) or UHV (gray). Fill colors represent the substituent cation: Ni (green), Zn (blue), increasing Al fraction (dark to light red) and LC (black). Lines added to guide the reader (top) or represent least squares fits (bottom).

Supplementary Note 5: Analysis of oxygen surface species

O 1s core electron spectra (Supplementary Figures 7 and 8) were aligned and normalized to the bulk oxide species in terms of position (528.9 eV) and height, respectively. Peaks related to surface species of hydroxyl, carbonate groups or surface oxygen are observed in a range of 530-534 eV in line with common findings for perovskites [9,10]. Hydroxyl groups are attributed to the peak 530.5 eV [9] but the origin of the other two peaks related to surface species is ambiguous and cannot be discerned between surface oxygen and carbonate surface in absence of respective C 1s spectra. An additional species with a peak center at 534.7 - 535.7 eV appears for the catalysts LCZ-82 and LCA-82. The latter was analyzed in detail for a set of different conditions The elevated binding energy of the peak implicates a surface species that is less negatively charged than the other surface species, possibly a superoxide-type species. It is important to note that peaks at this binding energy have been related to O in fluorinated hydrocarbons that are a potential surface impurity[11]. In some measurements respective peaks[12] in C 1s (291-293 eV) and a F 1s signal is present in the survey measurement in few instances. The C1s and F 1s peaks, however, do not occur consistently with the O 1s species at elevated binding energy. Vice versa, catalysts that show the presence of fluorinated hydrocarbons in C1s and F 1s do not show the peak in O 1s that we ascribe to superoxide species. As a result, we ascribe the peak above 534 eV to a superoxide species. Additional experiments to confirm this designation are in line.

Species above 534 eV have also been attributed to liquid phase H_2O[13,14], which was generated through an experimental setup that was not present in our study and, thus, liquid phase H_2O is very unlikely to be the origin of the peak in question. Original publications that were cited to attribute the species above 534 eV to adsorbed water in references[15,16] do not distinctly describe adsorbed water at these binding energies; instead adsorbed H_2O is ascribed to peaks below 534 eV in other reports[9,10].

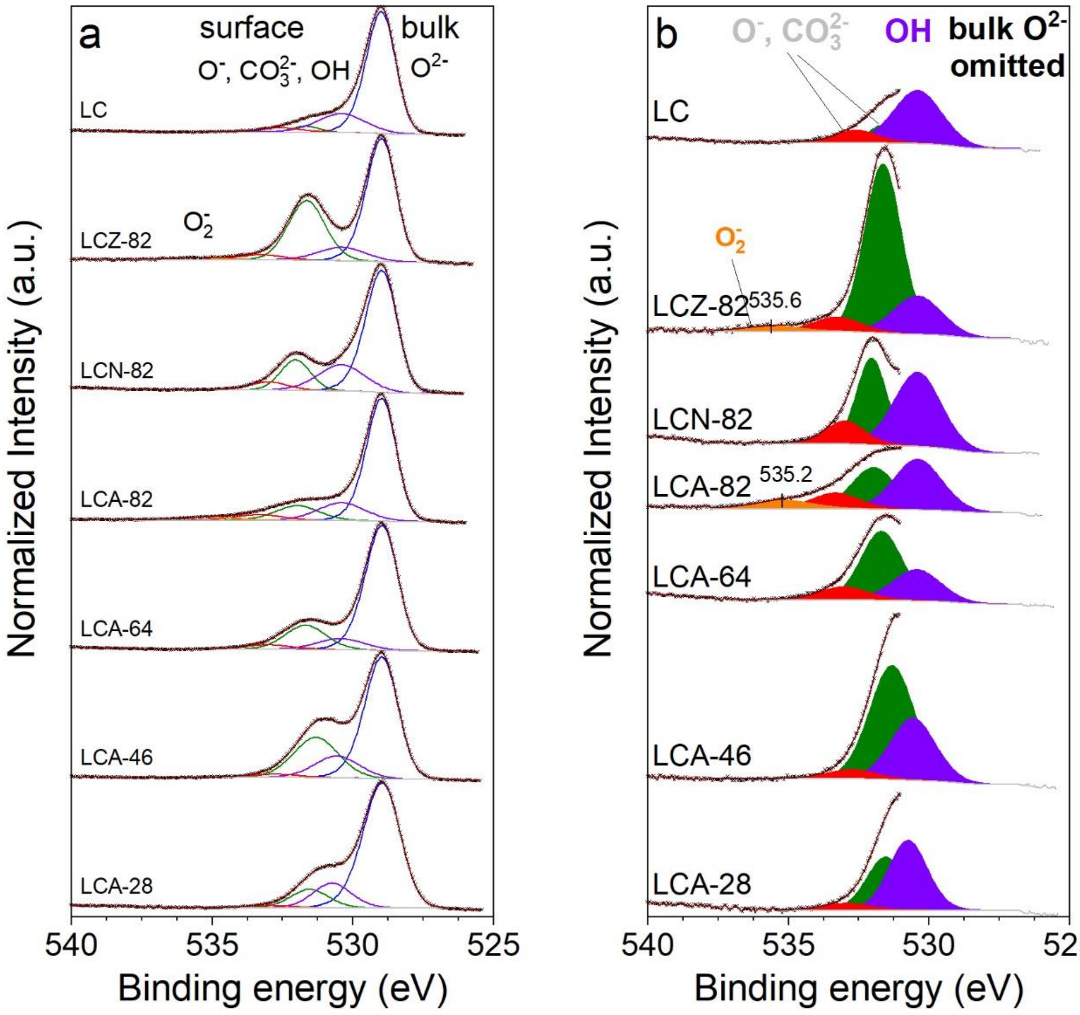

Supplementary Fig. 7 Comparison of O 1s spectra of catalysts in UHV at 623 K **a** Full spectra and components. **b** Fit of the same spectra of LCA-82 in a more detailed illustration of the peaks related to oxygen surface species, where O^- and CO_3^{2-} were not distinguishable in terms of peak attribution.

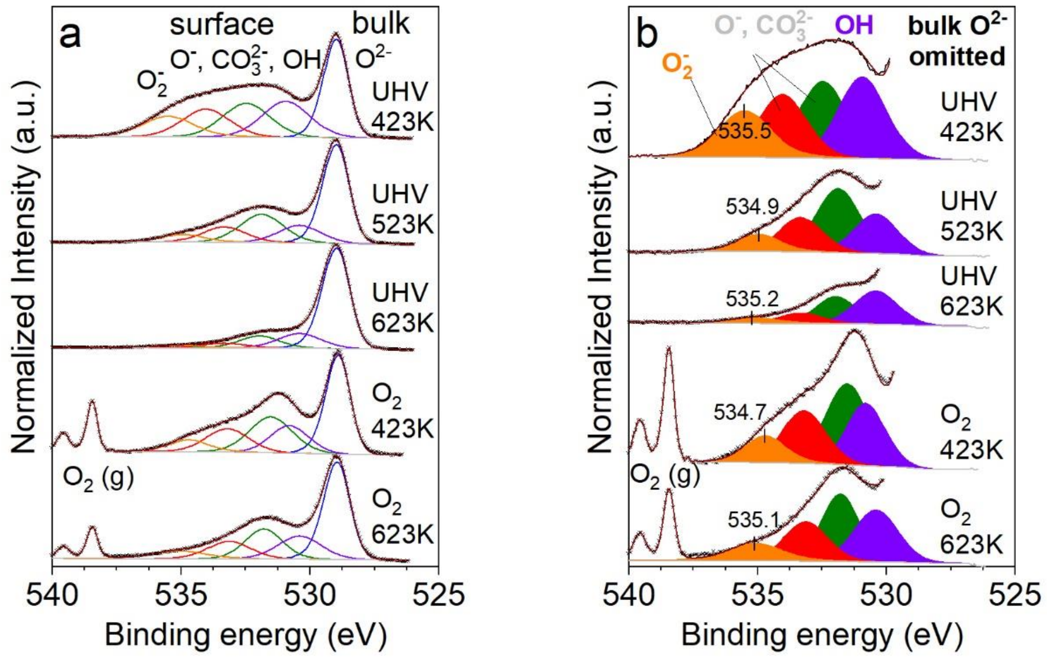

Supplementary Fig. 8 Comparison of O 1s spectra of $LaCo_{0.8}Al_{0.2}O_3$ (LCA-82) in different experimental conditions. **a** Full scale of spectra and components**. b** Fit of the same spectra of LCA-82 in a more detailed illustration of the peaks related to oxygen surface species, where O^- and CO_3^{2-} were not distinguishable in terms of peak attribution.

Supplementary Note 6: Partial Pressure Dependence in CO Oxidation

The trend of CO oxidation with partial pressures was tested to confirm previous mechanistic assumptions for our study: Figure S8 shows similar trends of reactant partial pressures as described previously[17], where CO and O_2 partial pressures have a favorable effect on the CO oxidation rate, while increasing CO_2 partial pressure has the inverse effect. CO and CO_2 adsorb on the same O surface sites[17]. Thus, CO_2 partially inhibits CO adsorption and its subsequent reaction. The increase of CO partial pressure accordingly leads to an increase in CO oxidation rate as its concentration on the surface increases in the competitive adsorption regime. As a result, we assume the mechanism described previously[17] is valid for the discussions in our work as well.

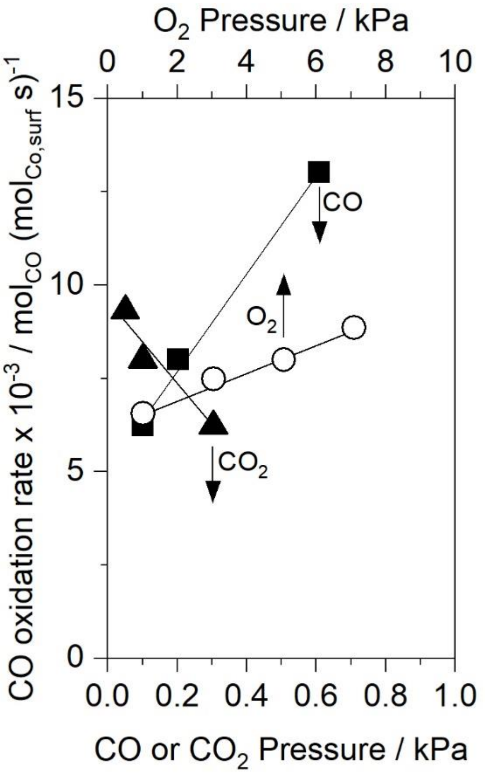

Supplementary Fig. 9 Effect of reactant partial pressure on CO oxidation rates. Reaction rates measured on LC at 519 K. Lines were added to guide the reader.

Supplementary Table 5 Gas concentrations in rate measurements and information on gas supply

	NO oxidation			CO ox. or N_2O decomp.				Inert Gases		
Gas	NO	NO_2	O_2	CO	CO_2	O_2	N_2O	He	Ar	N_2
Concentration (%) in gas cylinder (Purity)	2.95 (1.8)	1.00 (2.0)	19.95 (3.8)	1.0 (2.8)	20.5 (2.8)	20.5 (4.0)	1.0 (2.5)	100 (5.0)	100 (4.8)	100 (5.0)
Supplier*	Pr	Pr	Pr	Pr	Wf	Wf	Pr	Pr	Wf	Wf

*Gas suppliers were Westfalen GmbH (Wf) and Praxair (Pr).

Supplementary References

1 Aswin, V. *et al.* Influence of Al doping in LaCoO3 on structural, electrical and magnetic properties. *J. Mater. Sci.* **50**, 366-373 (2015).

2 Ghiasi, M. *et al.* Mn and Co Charge and Spin Evolutions in LaMn1–x Co x O3 Nanoparticles. *J. Phys. Chem. C* **120**, 8167-8174 (2016).

3 Haverkort, M. W. *et al.* Spin State Transition in LaCoO3 Studied Using Soft X-ray Absorption Spectroscopy and Magnetic Circular Dichroism. *Phys. Rev. Lett.* **97**, 176405 (2006).

4 Petrie, J. R. *et al.* Enhanced Bifunctional Oxygen Catalysis in Strained LaNiO3 Perovskites. *J. Am. Chem. Soc.* **138**, 2488-2491 (2016).

5 Abbate, M., Potze, R., Sawatzky, G. A. & Fujimori, A. Band-structure and cluster-model calculations of LaCoO3 in the low-spin phase. *Phys. Rev. B* **49**, 7210-7218 (1994).

6 Suntivich, J. *et al.* Estimating Hybridization of Transition Metal and Oxygen States in Perovskites from O K-edge X-ray Absorption Spectroscopy. *J. Phys. Chem. C* **118**, 1856-1863 (2014).

7 Palina, N. *et al.* Electronic defect states at the LaAlO3/SrTiO3 heterointerface revealed by O K-edge X-ray absorption spectroscopy. *PCCP* **18**, 13844-13851 (2016).

8 Hong, W. T. *et al.* Probing LaMO3 Metal and Oxygen Partial Density of States Using X-ray Emission, Absorption, and Photoelectron Spectroscopy. *J. Phys. Chem. C* **119**, 2063-2072 (2015).

9 Stoerzinger, K. A. *et al.* Water Reactivity on the LaCoO3 (001) Surface: An Ambient Pressure X-ray Photoelectron Spectroscopy Study. *J. Phys. Chem. C* **118**, 19733-19741 (2014).

10 González Tejuca, L., Bell, A. T., Fierro, J. L. G. & Peña, M. A. Surface behaviour of reduced LaCoO3 as studied by TPD of CO, CO2 and H2 probes and by XPS. *Appl. Surf. Sci.* **31**, 301-316 (1988).

11 Schulze, M., Lorenz, M., Wagner, N. & Gülzow, E. XPS analysis of the degradation of Nafion. *Fresenius J. Anal. Chem.* **365**, 106-113 (1999).

12 Lei, Y.-G., Ng, K.-M., Weng, L.-T., Chan, C.-M. & Li, L. XPS C 1s binding energies for fluorocarbon–hydrocarbon microblock copolymers. *Surf. Interface Anal.* **35**, 852-855 (2003).

13 Lichterman, M. F. *et al.* Direct observation of the energetics at a semiconductor/liquid junction by operando X-ray photoelectron spectroscopy. *Energy Environ. Sci.* **8**, 2409-2416 (2015).

14 Karslıoğlu, O. *et al.* Aqueous solution/metal interfaces investigated in operando by photoelectron spectroscopy. *Faraday Discuss.* **180**, 35-53 (2015).

15 Ponce, S., Peña, M. A. & Fierro, J. L. G. Surface properties and catalytic performance in methane combustion of Sr-substituted lanthanum manganites. *Appl. Catal. B* **24**, 193-205 (2000).

16 Machocki, A. *et al.* Manganese–lanthanum oxides modified with silver for the catalytic combustion of methane. *J. Catal.* **227**, 282-296 (2004).

17 Tascón, J. M. D., Fierro, J. L. G. & Tejuca, L. G. Kinetics and Mechanism of CO Oxidation on LaCoO3. *Z. Phys. Chem.* **124**, 249 (1981).
